# Supplementary material for: Piezo1 balances membrane and cortex tension to stabilize intercellular junctions and maintain the epithelial barrier
Source: J Cell Sci. 2025 Sep 1;138(16):jcs263938. doi: 10.1242/jcs.263938 (PMC12450467; doi:10.1242/jcs.263938)
Supplement: Supplementary information [file joces-138-263938-s1.pdf]

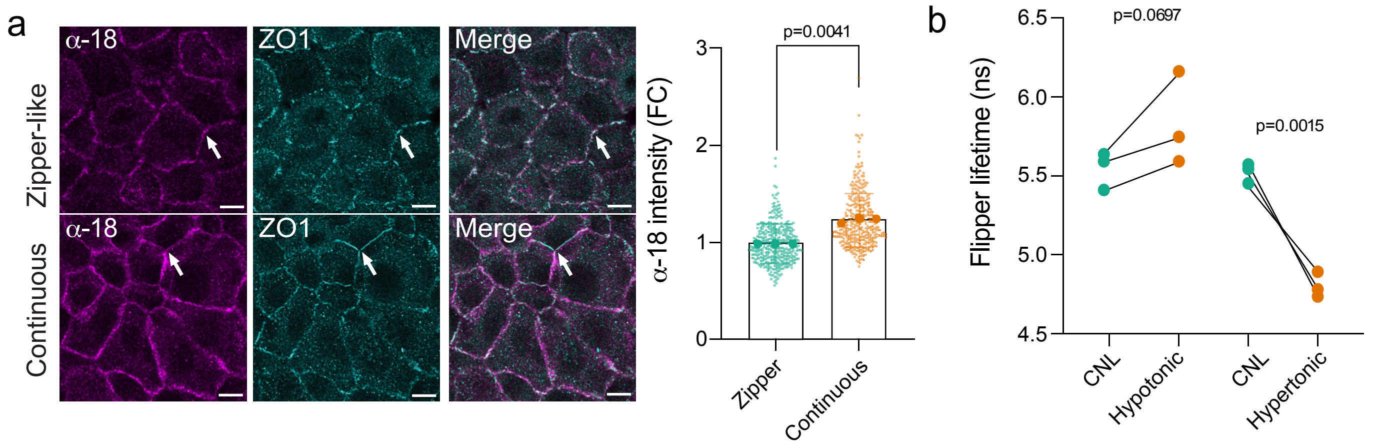

Fig. S1. Analyses of junction and membrane tension after calcium switch or osmotic shocks

a) Representative images and quantification of tension-sensitive α-catenin (α-18) antibody and ZO-1-stained primary keratinocytes fixed at 4 h or 8 h post Ca^{2+} addition. Arrows indicate zipper-like and continuous junctions. Note the increase of α-18 intensity at continuous junctions (n=3 independent experiments with >75 cells/experiment/condition; mean±SD; Wilcoxon test; scale bar 10 µm). **b)** Quantification of fluorescence lifetime imaging of FLIPPER-TR from hypotonic and hypertonic controls (added 1 min before measurements) in primary keratinocytes (n=3 independent experiments with >10 membrane measurements per condition/experiment).

Journal of Cell Science • Supplementary information

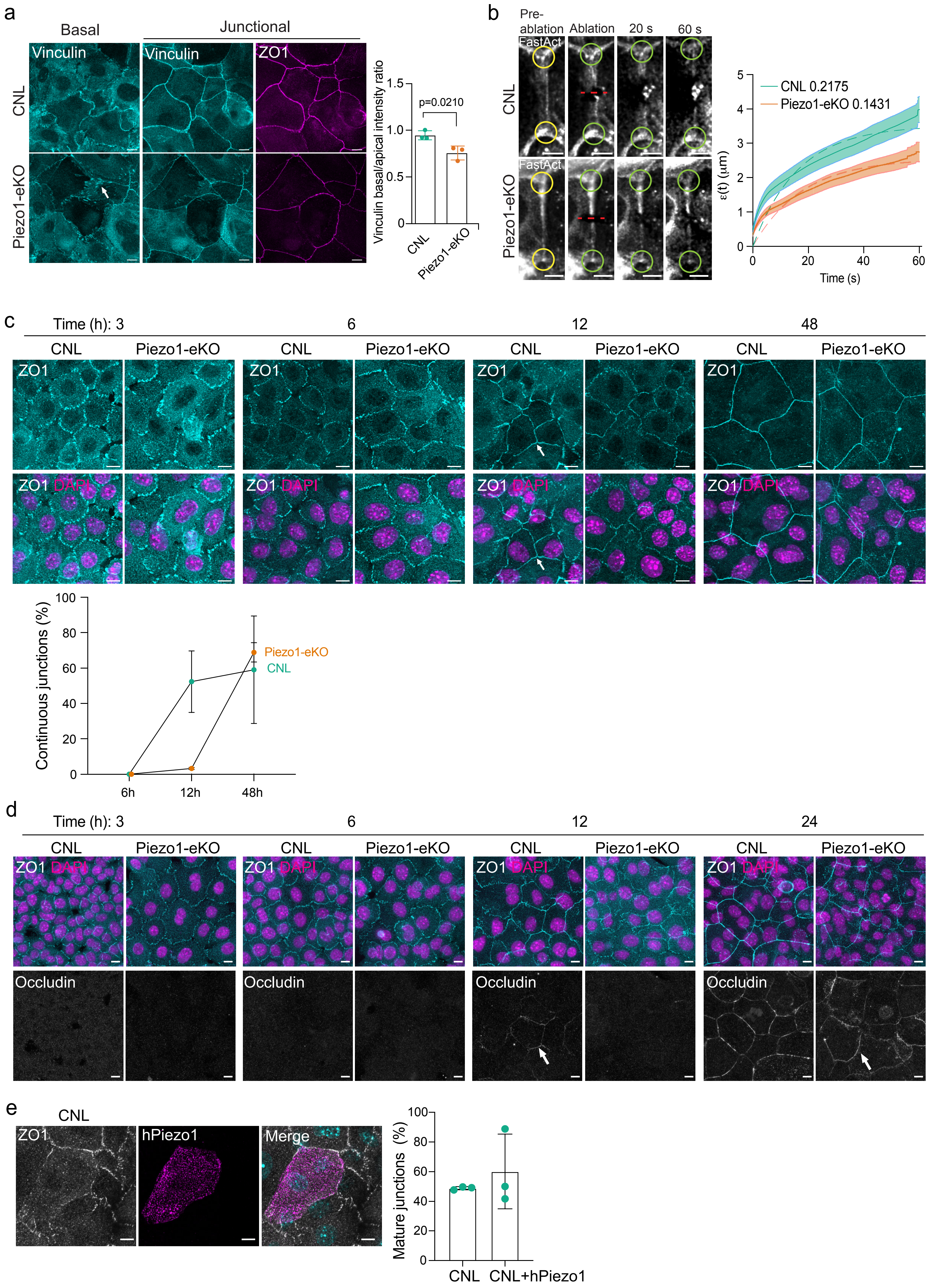
a
Basal
Junctional
Vinculin
Vinculin
ZO1
CNL
Piezo1-eKO
Vinculin basal/apical intensity ratio
p=0.0210
1.5
1.0
0.5
0
CNL
Piezo1-eKO
b
Pre-ablation
Ablation
20 s
60 s
FastAct
CNL
Piezo1-eKO
CNL 0.2175
Piezo1-eKO 0.1431
ε(t) (μm)
Time (s)
c
Time (h): 3
6
12
48
CNL
Piezo1-eKO
ZO1
ZO1 DAPI
Continuous junctions (%)
Piezo1-eKO
CNL
6h
12h
48h
d
Time (h): 3
6
12
24
CNL
Piezo1-eKO
ZO1 DAPI
Occludin
e
CNL
ZO1
hPiezo1
Merge
Mature junctions (%)
CNL
CNL+hPiezo1

Fig. S2. Piezo1-eKO cells show adhesion maturation defects

a) Representative images of vinculin- and ZO-1-stained CNL and Piezo1-eKO primary keratinocytes fixed 24 h post Ca^{2+} switch. Quantification shows increased intensity of vinculin (arrow) in Piezo1-eKO basal focal adhesion plane compared to apical junctional plane (n =3 independent experiments with >70 cells/experiment/condition; unpaired t-test; scale bars 10µm). **b)** Representative images and quantification of FastAct-labeled keratinocytes subjected to laser ablation 8 h post Ca^{2+} switch. Quantification shows attenuated recoil velocity of Piezo1-eKO vertexes (marked by circles) post ablation indicative of decreased junctional tension (n = 32 cells/ condition pooled across 3 independent experiments; Lowess fit; scale bars 10µm). **c)** Representative images and quantification of ZO-1- and DAPI-stained primary keratinocytes fixed at indicated time points post Ca^{2+} switch. Quantification shows delayed junction maturation of ZO-1 junctions in Piezo1-eKO cells (n=3 independent experiments with >100 cells/experiment/condition; mean±SD; scale bar 10 µm). **d)** ZO-1, DAPI- and occludin-stained primary keratinocytes fixed at indicated time points post Ca^{2+} switch. Arrows indicate occludin-positive junctions. Note delayed emergence of occludin-positive tight junctions in Piezo1-eKO cells (n=3 independent experiments with >40 cells/experiment/condition; mean±SD; scale bar 10 µm). **e)** Representative images and quantification of junction maturation in CNL keratinocytes transfected with hPiezo1 and fixed 8h post Ca^{2+} switch. Overexpression of Piezo1 in CNL cells does not impact junction maturation (n=3 independent experiments with >60 cells/experiment/condition; mean±SD; scale bar 10 µm).

Journal of Cell Science • Supplementary information

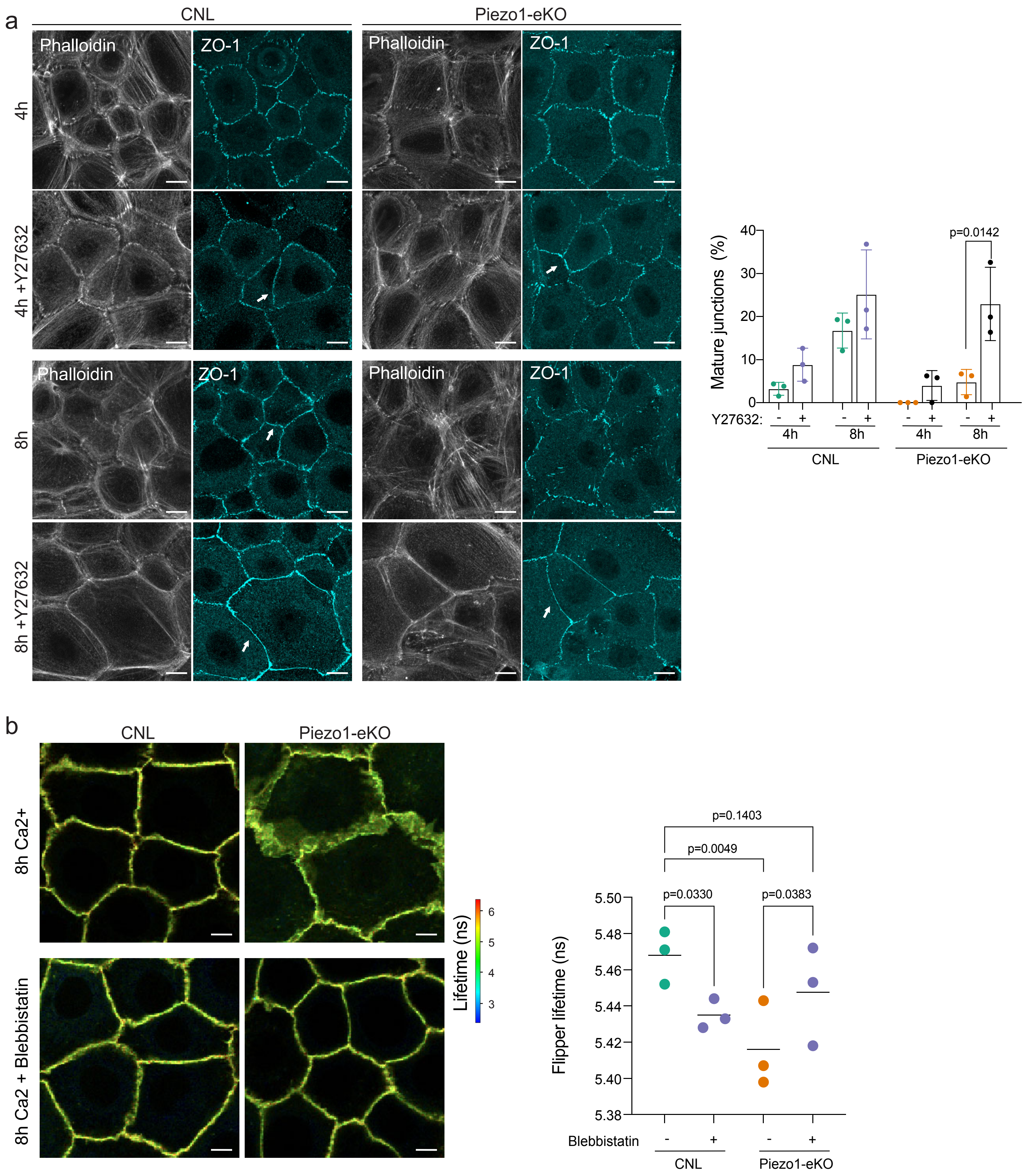

Fig. S3. Impact of actomyosin contractility on junction maturation and membrane tension

a) Representative images and quantification of ZO-1- and phalloidin-stained primary keratinocytes fixed at indicated time points post Ca^{2+} switch. Arrows show ZO-1 in mature junctions. Quantification shows delayed junction maturation of ZO-1 junctions in Piezo1-eKO cells and rescue with blebbistatin treatment (n=3 independent experiments with >30 cells/experiment/condition; mean±SD; scale bar 10 µm). **b)** Representative images and quantification of fluorescence lifetime imaging of FLIPPER-TR. Note decrease in FLIPPER-TR lifetime indicative of lowered membrane tension in Piezo1-eKO cells after 8 h Ca^{2+} and restoration of lifetime when blebbistatin is added to cells for the last 2h before measurement (n=3 independent experiments with ≥ 50 membrane measurements per condition/experiment; RM-ANOVA/Fischer's; scale bar 10 µm).

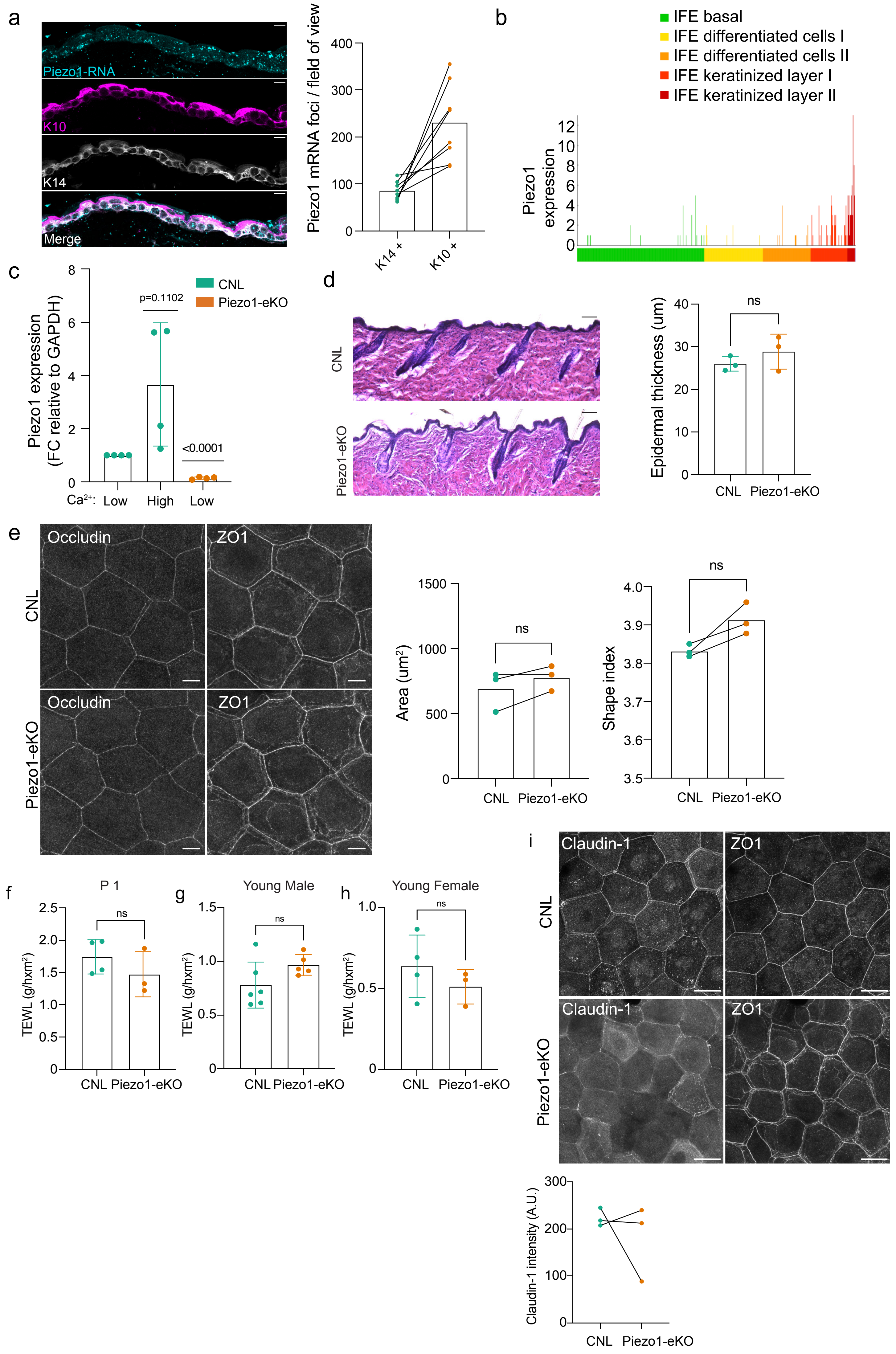

Journal of Cell Science • Supplementary information

Fig. S4. Analyses of Piezo1 expression and epidermal phenotype in young Piezo1-eKO mice

a) Representative images of 1-y old mouse back skin stained for Piezo1-RNA, Keratin-10 (K10) and Keratin-14 (K14) (n=8 fields of view). **b)** Analysis of Piezo1 mRNA expression from single cell RNAseq data from adult mouse back skin shows most abundant mRNA levels in suprabasal layers [51]. **c)** RT-qPCR shows increased expression of Piezo1 in cells cultured under high Ca^{2+} calcium (n = 4 independent experiment, Kruskal-Wallis/Dunn's). **d)** Representative H/E-stained back skin sections of CNL and Piezo1-eKO back skin mice (3-6 months old). Quantification comparable epidermal thickness between genotypes (n= 3 mice/genotype; unpaired t-test; scale bar 100 µm). **e)** Representative images and quantification of cell area and shape of ZO-1-stained ear whole mounts from 3-month-old CNL and Piezo1-eKO mice. (n =3 mice / genotype, paired t-test, scale bar 10 µm). **f)** Quantifications of transepidermal water loss measurements from back skin of CNL and Piezo1-eKO at postnatal day 1 (P1) (n=4 (CNL) and 3 (Piezo1-eKO) mice; unpaired t-test). **g)** Quantifications of transepidermal water loss measurements from back skin of 3 months old male CNL and Piezo1-eKO (n=6 (CNL) and 5(Piezo1-eKO) mice; unpaired t-test. **h)** Quantifications of transepidermal water loss measurements from back skin of 3 months old female CNL and Piezo1-eKO (n=4 (CNL) and 3 (Piezo1-eKO) mice; unpaired t-test). ns=not significant. **i)** Representative images and quantification of claudin-1 staining from ear whole mount images of CNL and Piezo1-eKO 3 month-old mice (n=3 mice/genotype; paired t-test, scale bars 10µm). A.U.=arbitrary units.

Journal of Cell Science • Supplementary information

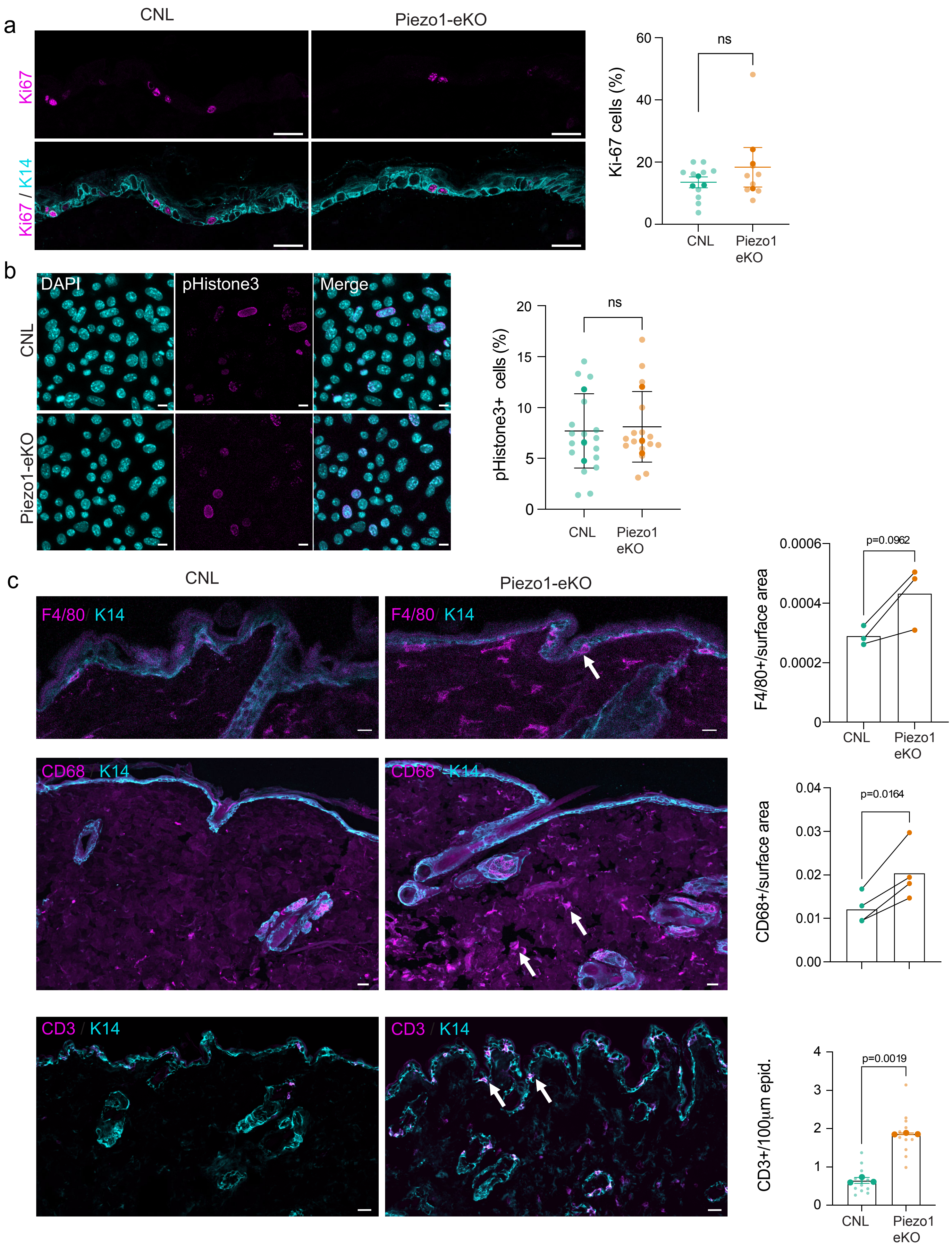

Journal of Cell Science • Supplementary information

Fig. S5. Analyses of epidermal phenotype of aged Piezo1-eKO mice

a) Representative images and quantification of CNL and Piezo1-eKO mice (young 3-6 months old) back skin stained for Ki-67 (magenta) and Keratin-14 (K14; cyan). Quantifications show no significant difference in Ki-67 levels between CNL and Piezo1-eKO mice (n=3 mice / genotype, Mann-Whitney, scale bar 20 µm). **b)** Representative images and quantification of phosphorylated Histone H3 (H3P)-stained CNL and Piezo1-eKO cultured primary keratinocytes. No differences in proliferation were observed *in vitro* (n=3 independent experiments; paired t-test; scale bar 10 µm). **c)** Representative images and quantification of CNL and Piezo1-eKO <1-year old mice back skin stained for F4/80, CD68 and CD3 (magenta) and Keratin-14 (K14; cyan). Note increased presence of immune cells (arrows) in the skin of Piezo1-eKO mice (n=3-4 mice/genotype; paired t-test; scale bar: F4/80, 10 µm; CD3 and CD68, 20 µm). ns= not significant.

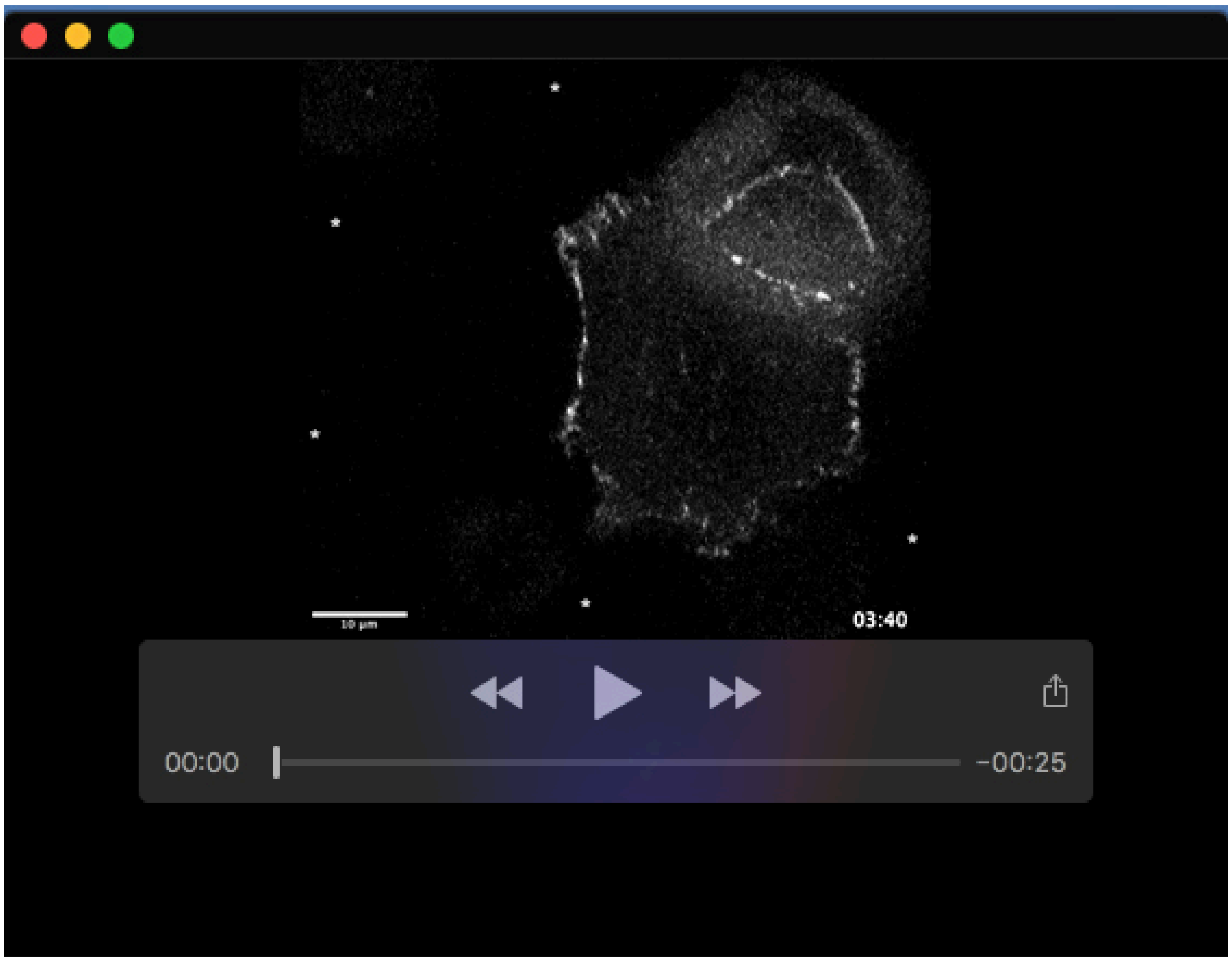

Movie 1. Analysis of adhesion maturation upon calcium addition in cultured primary keratinocytes

Live imaging of keratinocytes transfected with ZO-1-mEmerald. Note the transition of zipper-like adherens junctions into continuous, “belt-like” junctions starting around 5 h post Ca^{2+} switch. Arrow indicates the mature belt-like junction, asterisks mark untransfected neighboring cells. Acquisitions were performed at a rate of 20 min frame^{-1} for 16 h. Scale bars, 10 µm.

Journal of Cell Science • Supplementary information

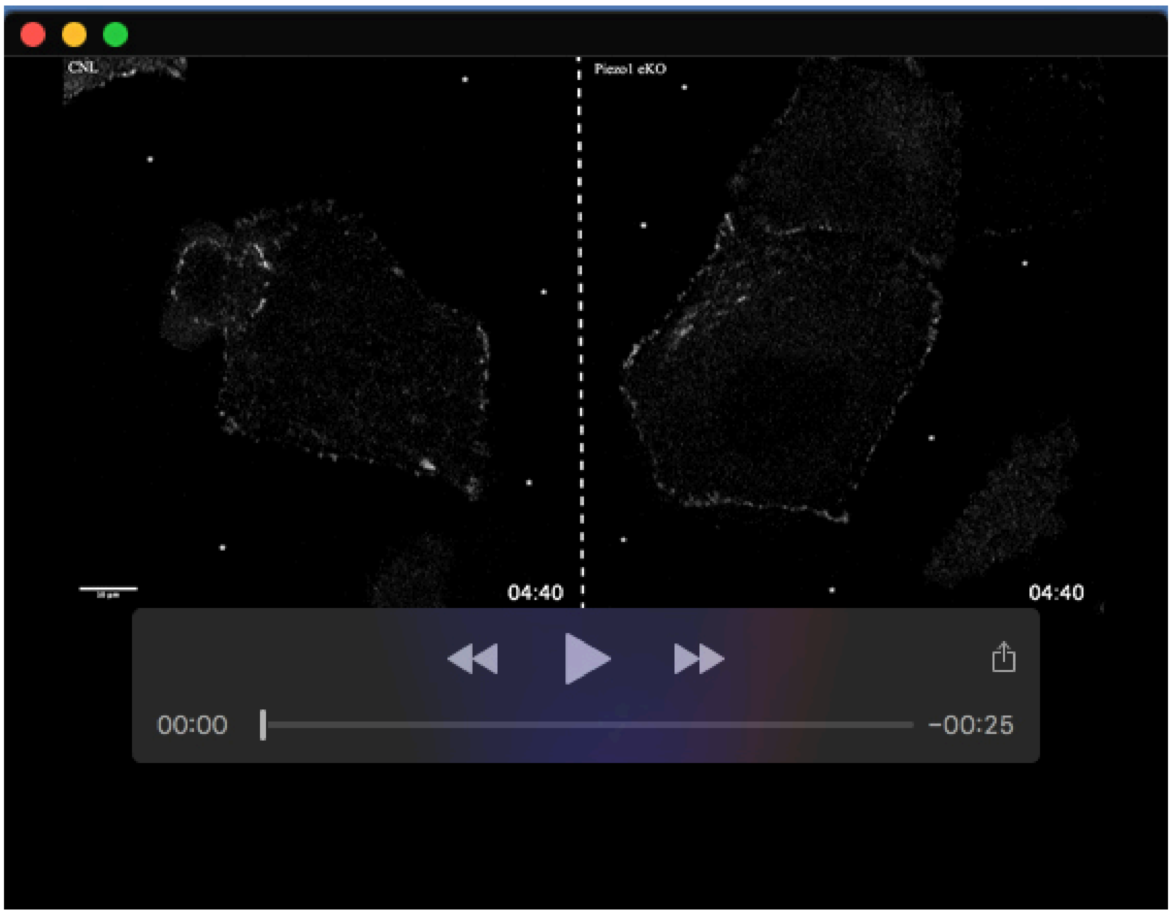

Movie 2. Comparison of adhesion maturation between CNL and Piezo1-eKO keratinocytes

Live imaging of cultured CNL and Piezo1-eKO keratinocytes transfected with ZO-1-mEmerald. Note the faster transition of zipper-like junctions into continuous junctions in CNL cell (left) post Ca^{2+} switch. Arrow indicates the maturing mature belt-like junction, asterisks mark non-transfected neighboring cells. Acquisitions were performed at a rate of 20 min frame^{-1} for 16 h. Scale bars, 10 μm.

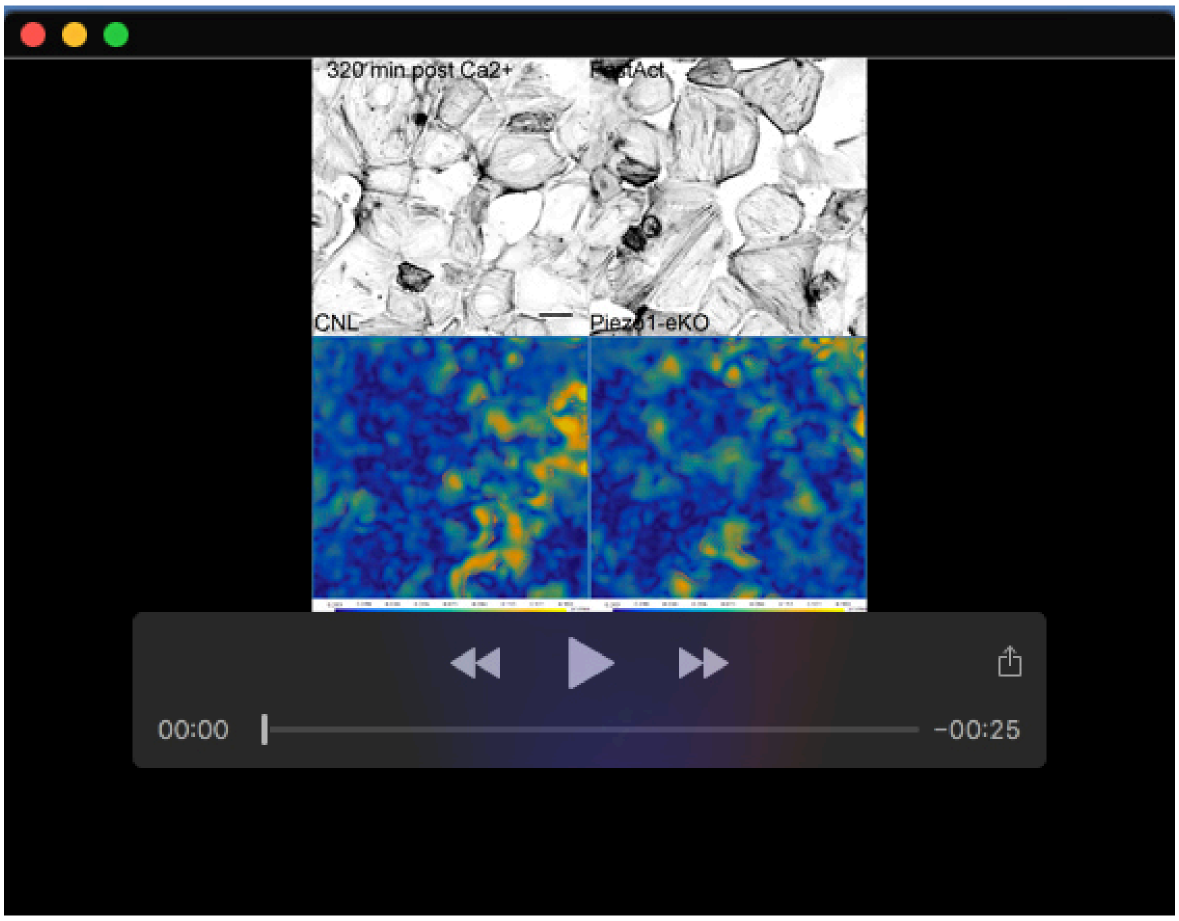

Movie 3. Comparison of monolayer motility dynamics between CNL and Piezo1-eKO keratinocytes

Live imaging of cultured CNL and Piezo1-eKO keratinocytes labeled with FastAct to visualize actin (upper panel) and the corresponding PIV quantifications of motion velocities. Note more dynamic behavior of Piezo1-eKO keratinocytes. Acquisitions were performed at a rate of 20 min frame^{-1} for 16 h. Scale bars, 10 μm.

Journal of Cell Science • Supplementary information
